# Torque teno virus load as marker of rejection and infection in solid organ transplantation – A systematic review and meta‐analysis

**DOI:** 10.1002/rmv.2393

**Published:** 2022-09-03

**Authors:** AL van Rijn, R Roos, FW Dekker, JI Rotmans, MCW Feltkamp

**Affiliations:** ^1^ Department of Medical Microbiology Leiden University Medical Center Leiden The Netherlands; ^2^ Department of Internal Medicine HagaZiekenhuis The Hague The Netherlands; ^3^ Department of Clinical Epidemiology Leiden University Medical Center Leiden The Netherlands; ^4^ Department of Internal Medicine Leiden University Medical Center Leiden The Netherlands

**Keywords:** biomarker, immunocompromised, solid organ transplantation, TTV

## Abstract

Balancing immunosuppression to prevent rejection in solid organ transplant (SOT) recipients remains challenging. Torque teno virus (TTV), a commensal non‐pathogenic virus, has been proposed as marker of functional immunity: higher loads correspond to over‐immunosuppression, and lower loads to under‐immunosuppression. This review offers an overview of the current evidence of the association between TTV‐load and infection and rejection after SOT. A systematic literature search strategy, deposited in the PROSPERO registry, resulted in 548 records. After screening, 23 original and peer‐reviewed articles were assessed investigating the association between TTV‐load, infection and/or rejection in SOT. The Quality in Prognostic Studies (QUIPS)‐tool was used to assess the risk of bias. Meta‐analysis with random‐effects was performed on results with similar outcomes and exposure measures. Most of the included studies involved retrospective cohorts in which the TTV‐load was measured longitudinally, within the first 2 years post‐transplantation. Infection outcomes differed between studies and included viral, bacterial, parasitic and fungal infections. Rejection was defined by biopsy confirmation or initiation of rejection treatment. Twelve out of 16 studies reported an association between high TTV‐load and infections, whereas 13 out of 15 reported an association between low TTV‐load and rejection. Meta‐analysis showed an increased risk of infection (OR: 1.16, 95% CI: 1.03–1.32; HR: 1.05, 95% CI: 0.97–1.14) and a decreased risk of rejection (OR: 0.90, 95% CI: 0.87–0.94; HR: 0.74, 95% CI: 0.71–0.76) per 1 log TTV‐load increase. The qualitative assessment showed varying risks of bias in the included studies. This systematic review and meta‐analysis indicates that blood TTV‐load measured within the first 2 years after SOT is associated with the risk of infection or allograft rejection, although substantial risk of bias in the studies included warrant cautious interpretation. The results in this review provide a rationale for larger, prospective, studies into TTV as marker of infection and rejection after SOT.

**Abbreviations**
ABMR: antibody mediated rejection
ACR: acute cellular rejection
ATG: anti‐thymocyte globulin
BKV: BK polyomavirus
BPR: biopsy proven rejection
CLAD: chronic allograft dysfunction
CMV: cytomegalovirus
HR: hazard ratio
KTx: kidney transplantation
LiTx: liver transplantation
LuTx: lung transplantation
OR: odds ratio
PBMC: peripheral blood mononuclear cell
PCR: olymerase chain reaction
QUIPS: quality in prognosis studies tool
SOT: solid organ transplantation
TTV: torque teno virus

## 1 INTRODUCTION

Post‐solid organ transplantation (SOT) care could substantially benefit from a marker that can measure immune function, so that immunosuppression can be reduced to a minimum needed to prevent graft rejection, while minimising the risk of infection. Thus far, it has proven difficult to anticipate and discriminate between complications due to over‐immunosuppression and under‐immunosuppression, as signs and symptoms frequently overlap. While current practice can quite adequately prevent rejection, the increased incidence of various infections and malignancies in the years following transplantation underlines the necessity to strive for the lowest‐limit of immune suppression, while assuring graft survival.

**FIGURE 1:**
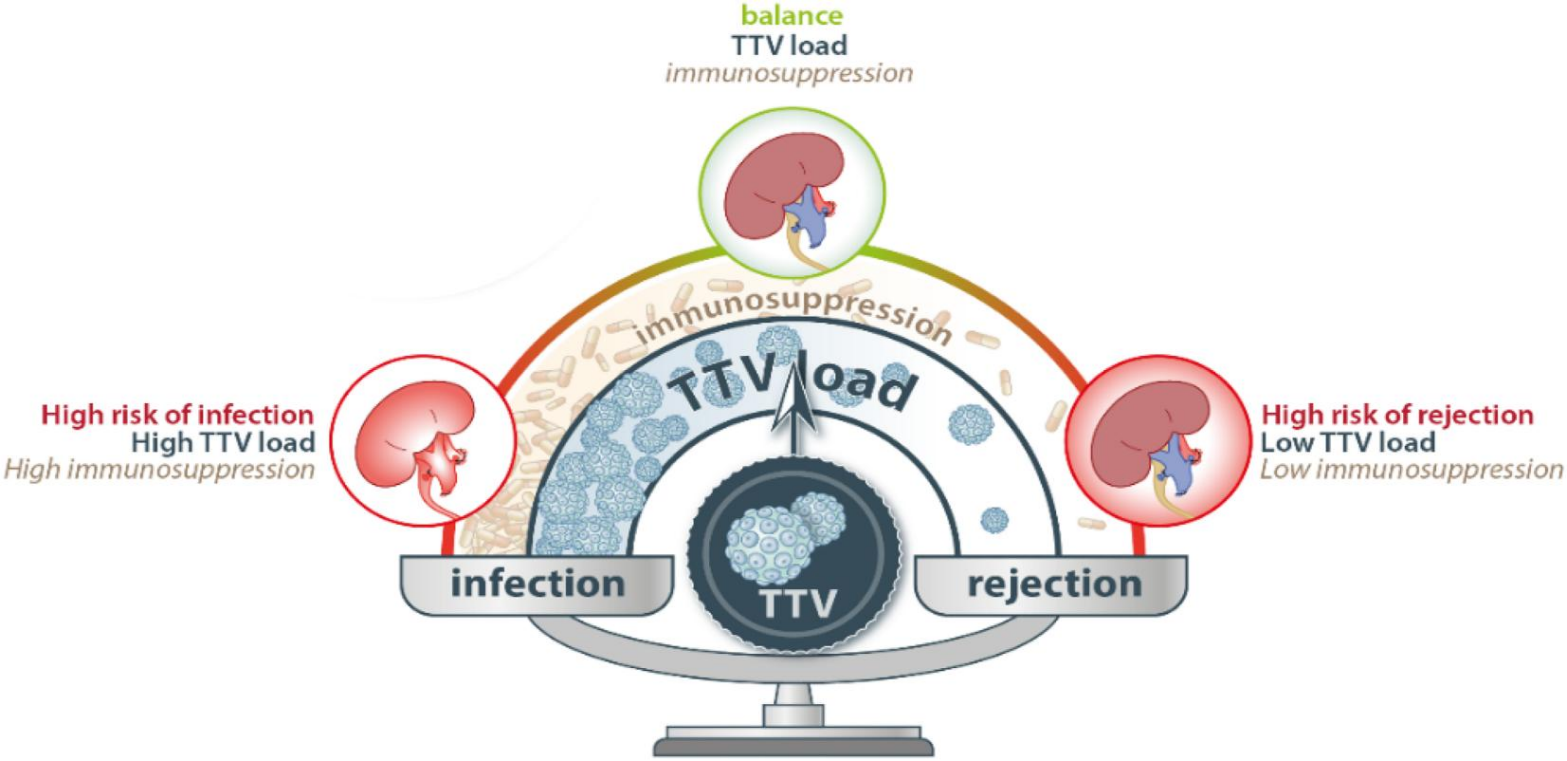
Graphical outline of TTV‐load as marker of infection and rejection

In recent years, Torque teno virus (TTV) has gained a lot of interest due to its potential to serve as a marker of immune function in SOT recipients.^1^, ^2^ TTV, a small single‐stranded DNA virus belonging to the *Anelloviridae*, is part of the human virome and without known pathogenicity. The load of this virus in blood is believed to reflect host immune function. For SOT recipients, this would mean that low or decreasing TTV‐load corresponds to under‐immunosuppression and therefore signal a higher risk of allograft rejection, and high or increasing TTV‐load corresponds to over‐immunosuppression and signal a higher risk of infectious complications (Figure 1). In clinical practice, therefore, the TTV‐load can potentially be used to predict increased risk of both rejection and infection, and to guide personalised immunosuppressive treatment regimens.

Multiple research groups have recently explored the association between TTV‐load and the complications of over‐ and under‐immunosuppression in different transplantation populations in detail, for example, the frequency of infection after lung transplantation (LuTx),^3^ of cytomegalovirus (CMV) infection and BK polyomavirus infection after kidney transplantation (KTx),^4^ and of allograft rejection after KTx.^4^, ^5^ These studies reported varying results using different methods. In this paper, we provide a systematic review of currently available studies on the relationship between TTV‐load and rejection and between TTV‐load and infection after SOT. We examine the results, perform a meta‐analysis for infection and rejection where possible, and discuss the implications for TTV load measurement in clinical practice.

## 2 METHODS

A systematic review of the literature was performed, in which the evidence on the association between TTV‐load and the development of infections and rejection after SOT was analysed. The study protocol for this systematic review was deposited and can be found on PROSPERO (CRD42020193090).^6^

### 2.1 Literature search

**FIGURE 2:**
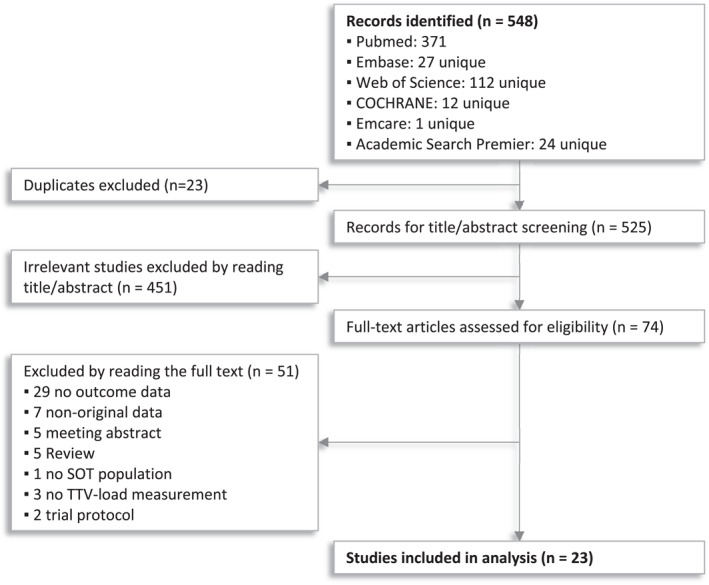
Flowchart of systematic literature search and screening

In consultation with a local information specialist, electronic databases including Medline, Embase, Web of Science, COCHRANE Library, Emcare and Academic Search Premier, were searched for relevant studies from articles published in English until 18‐06‐2021. A flowchart is provided in Figure 2. The full search strategy can be found in Supplementary S1. In brief, to identify studies eligible for inclusion, all identified records were screened on title/abstract, which was followed by full‐text screening of the selected records.

Literature screening was performed independently by two researchers (AvR and RR). Screening software Covidence was used to facilitate screening and remove duplicates. We included studies based on the following inclusion criteria: (1) population of SOT recipients, (2) TTV‐load measurement performed at least once, (3) data reports on infectious complications and/or allograft rejection. Reviews, meeting abstracts and studies with non‐original data were excluded. Doubts about article inclusion were resolved through discussion with a third researcher (MF).

### 2.2 Data extraction

Data extraction was performed independently and cross‐checked by two researchers (AvR and RR). The extracted data included: bibliographical information, study design and characteristics, exposure definition (primers and timepoint), outcome definition (including pathogen and timepoint), characteristics of study participants adult/pediatric, type of transplantation, immunossupressive regimen and number of participants and participants with outcome per group, and effect estimates with standard error, the latter derived from the confidence interval if necessary.

### 2.3 Risk of bias evaluation

Systematic appraisal of bias in the included studies was supported by the Quality in Prognosis Studies tool (QUIPS), which is a generally accepted checklist of prompting items and considerations identified and validated by a working group of epidemiologists, statisticians and clinicians, to aid researchers in judging the risk of bias in studies of prognostic factors.^7^

With the help of the QUIPS tool the risk of bias in the included studies was assessed over five domains (study participation, study attrition, prognostic factor measurement, outcome measurement, and analysis and reporting). The ‘confounding measurement and account’ domain was omitted, since the purpose of identifying the relationship between TTV‐load and outcomes is prediction, and not uncovering a causal effect.^8^ Assessment was performed independently by AvR and RR, and differences were resolved through discussion with MF.

### 2.4 Analysis

Studies were summarised by text and in evidence tables. The numeric results were reported from studies that estimated odds ratios (ORs) or hazard ratios (HRs). Studies that only reported mean differences in measured TTV‐load are summarised in text only. If ORs were not reported, but the predictive ability of a TTV‐load threshold was reported, ORs and corresponding confidence intervals were estimated. Meta‐analysis was performed for studies with identical exposure definition (TTV‐load increase as continuous variable, per 1 log) and a similar effect measure (OR or HR). Random‐effect models were used to account for heterogeneity, which was considered high due to the use of different analysis methods, outcomes, and timepoints on which the TTV‐load was measured. Pooling of ORs and HRs was performed with the DerSimonian & Laird method,^9^ and all analyses and corresponding visualisation was conducted using the R‐package ‘metafor’.^10^ The alpha level for statistical significance was set at 0.05.

When studies reported more than one analysis that could be included in meta‐analysis, only one was selected for inclusion to avoid duplication of subjects. This choice was based on the following considerations: for rejection, the main analysis was included, and for infection, the analysis with the most ‘inclusive’ definition of infection was included (usually a combination of infections of different origin). If possible, the crude analysis was included, since this enables more valid comparison, and the association of interest in not concerned with causality. Some studies used a ‘repeated measures’ analysis for the exposure (time‐varying covariate, joint models), in which case the effect from this analysis was included. Lastly, analyses with one event (outcome) per patient were preferred over analyses with multiple events per patient, without appropriate adjustment.

Residual heterogeneity was assessed using the Cochran Q‐statistic and the I^2^ statistic. Estimates with a *p*‐value 0.05 for the *Q*‐statistic and *I* ^2^ >50% were considered to have significant heterogeneity. To explore the effect of heterogeneous populations and methods, additional meta‐analyses were performed restricted to specific patient populations (only adult, only KTx recipients) or to specific TTV detection methods (use of common in‐house TTV primer set only). Assessment of publication bias through a funnel plot was considered if more than 10 studies were pooled in a meta‐analysis.

## 3 RESULTS

In total, 548 records were identified with the search strategy. After removing duplicates, screening titles and abstracts, and assessing full‐text articles, 23 studies were included for the systematic review (Figure 2).

### 3.1 Setting, transplant type, immunosuppression and prophylaxis

All included studies were based in Europe except for two studies from the USA.^11^, ^12^ SOT types included kidney (KTx), lung (LuTx) and liver (LiTx) (Table 1). Two studies included only pediatric patients, below 18 years of age,^11^, ^13^ and two included both adult and pediatric patients.^14^, ^15^ The remaining studies included only adult patients or did not report specific age criteria. KTx and LiTx studies that reported their immunosuppressive regimen all applied standard induction therapy: anti‐IL2 (basiliximab)/anti‐thymocyte globulin/none. Maintenance therapy consisted of either a triple or double immunosuppressive regimen, with calcineurin inhibitors (tacrolimus or cyclosporine A), and antimetabolites (mycophenolate mofetil, azathioprine) or mTOR‐inhibitors (everolimus or sirolimus), and corticosteroids, in different combinations. Immunosuppressive regimens for LuTx were higher in dosage but similar in type, with the addition of methylprednisolone as induction therapy in some centres. Most studies did not specifically report which antibacterial or antiviral prophylaxis was included in standard care, however, all antibacterial prophylaxis that was reported included trimethoprim and sulfamethoxazole. Antiviral prophylaxis included (val)ganciclovir, either for each patient or according to donor and recipient CMV serostatus. A detailed overview per study can be found in Supplementary S2.

**TABLE 1:** Overview of studies included in the systematic review

Original article				TTV PCR	Reported association^c^	
First author, year	Tx type^a^	Size^b^	Population	Primer set	Infection	Rejection
Blatter, ‘18	LuTx	57	Pediatric	Maggi et al.^22^		▼
Blatter, ‘20	LuTx	64	Adults	Maggi et al.		**=**
Doberer, ‘19	KTx	386	Adults	Maggi et al.	▲	▼
Doberer, ‘20	KTx	307	Adults	Maggi et al.		▼
Fernández‐Ruiz, ‘19	KTx	221	Adults	TTV R‐GENE^®^	▲	▼
Fernández‐Ruiz, ‘20	KTx	215	Adults	TTV R‐GENE^®^	▲	
Frye, ‘19	LuTx	34	n.m.^d^	Maggi et al.	▲	▼
Gore, ‘20	KTx	666	Adults	TTV R‐GENE^®^	▲	
Görzer, ‘14	LuTx	31	Both	Maggi et al.	▲	
Görzer, ‘17	LuTx	20	Adults	Maggi et al.		▼
Handala, ‘19	KTx	116	Adults	TTV R‐GENE^®^	**=**	
Herrmann, ‘18	LiTx	136	Adults	Maggi et al.	▲	
Jaksch, ‘18	LuTx	143	Adults	Maggi et al.	▲	▼
Maggi, ‘18	KTx + LiTx	280	Adults	Maggi et al.	▲	
Nordén, ‘17	LuTx	98	Adults	Maggi et al.	**=**	**=**
Ruiz, ‘19	LiTx	63	Adults	Maggi et al.	▲	▼
Schiemann, ‘17	KTx	715	Adults	Maggi et al.		▼
Simonetta, ‘17	LiTx	39	Both	Maggi et al.		▼
Solis, ‘19	KTx	66	Adults	TTV R‐GENE^®^	▲	▼
Strassl, ‘18	KTx	169	Adults	Maggi et al.	▲	
Strassl, ‘19	KTx	113	Adults	Maggi et al.		▼
Uhl, ‘20	KTx	45	Pediatric	Maggi et al.	**=**	
Van Rijn, ‘21	KTx	389	Adults	Maggi et al.	**=**	▼

*Note*: ▲ Authors conclude there is an association between high TTV‐load and the outcome. ▼ Authors conclude there is an association between low TTV‐load and the outcome. **=**Authors conclude there is no association.

^a^ *Tx: transplantation, LuTx: lung transplantation, KTx: kidney transplantation, LiTx: liver transplantation*.

^b^ Reported sample size. Actual sample size in the analyses may be different.

^c^ Whether the association between TTV‐loads and the outcome (meaningfully) exists according to authors.

^d^ n.m.: not mentioned.

### 3.2 Study designs and TTV‐load measurement

All included studies were observational, non‐interventional studies. Most consisted of cohorts (or cross‐sections thereof) of transplant recipients included at the time of transplantation,^13^, ^16^, ^17^, ^18^, ^19^, ^20^ while one study included patients who visited the hospital during a certain period.^21^ The time since transplantation at which the TTV‐load was measured differed across studies, and many repeated their analyses at different timepoints. As per inclusion criteria, all studies detected and quantified TTV by PCR, and estimated the genome copy number per millilitre of blood serum or plasma. Most studies used TTV‐primers and probes previously described by Maggi et al,^22^ that target a highly conserved untranslated region in the TTV‐genome and therefore detect a wide range of TTV‐genomes. Five more recent studies^21^, ^23^, ^24^, ^25^, ^26^ used the TTV R‐GENE® PCR assay, which also targets the untranslated region of the TTV‐genome and performs comparably to the assay described by Maggi et al., which was used as in‐house comparison.^21^, ^23^, ^24^, ^25^, ^26^, ^27^

### 3.3 Risk of bias assessment

**FIGURE 3:**
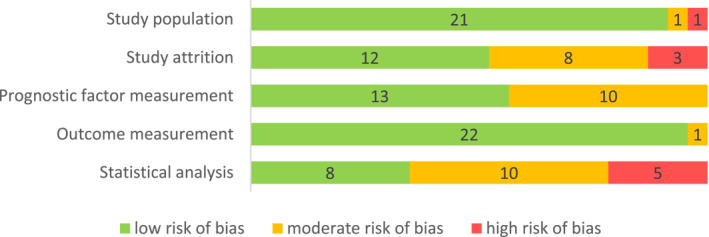
Risk of bias assessment. A total of 23 included studies were systematically assessed for publication bias over five domains listed on the left, with the help of the QUIPS tool

The risk of bias was systematically assessed independently by two reviewers with the help of the QUIPS‐tool^7^, and scored as low, moderate, or high across five domains (Figure 3). Most studies scored low in the ‘Study population’ domain, while two studies scored moderate or high because of uncertainty regarding the time period that was used to sample the study population.^16^, ^26^ Several studies had a moderate or high risk of bias in the ‘Study attrition’ domain, based on the way missing data and lost to follow‐up data were handled, as many studies simply excluded patients with missing data. For the ‘Prognostic factor measurement’ domain, the study scored a low risk of bias if the TTV‐load was measured in the same way for all patients. The risk of bias on this domain was scored as moderate for ten studies, because either the timing in measurement was different between groups, or the division of groups was data‐driven, or it was unclear whether an appropriate number of patients provided complete data on every timepoint. In the last domain ‘Statistical analysis’, many studies were classified as having a moderate or high risk of bias. Common sources of bias were selective reporting, especially for non‐significant results (‘data not significant, analysis not shown’), covariate selection based on confounding, violation of the independent observations assumption by incorporating multiple TTV‐load measurements per patient, not showing the same analyses for similar outcomes, and introducing immortal time bias by using TTV‐load measurements after time 0. Taken together, in a considerable number of studies we observed bias regarding study attrition and prognostic factor measurement and especially regarding statical analysis and reporting (Figure 3).

### 3.4 TTV‐load as marker of infection

Of all included studies, 16 studied infectious outcomes (Table 2).^3^, ^4^, ^13^, ^14^, ^16^, ^17^, ^21^, ^23^, ^25^, ^26^, ^28^, ^29^, ^30^, ^31^, ^32^ Main infectious outcomes ranged from composite outcomes– including viral, bacterial and fungal infections,^3^, ^13^, ^14^, ^28^, ^30^, ^31^, ^32^ and also parasitic infections in one study^23^—to specific outcomes such as for CMV^4^, ^17^, ^29^ or BK polyomavirus (BKV).^4^, ^16^, ^24^, ^26^ Several studies reported additional analyses for different types of infection. Definitions of ‘infection’ differed between studies, as many centres used the readily available information from patient records, for example, infections that caused hospitalisation, required treatment, were detected through standard monitoring, or caused death.

**TABLE 2:** Summary of evidence – infection outcomes

Study	Cases/total	Outcome^a^	TTV timepoint	Exposure^b^	OR/HR^c^		Other findings
Doberer, '19	127/274	V/B/F	±1 mo pre infection	C, 1	1.10	(1.02–1.17)^d^	Similar for infections not requiring hospitalisation, BKV, CMV, opportunistic and extra cellular bacterial infections.
Görzer, '14	13/24	V/B/F	28–76 days pre infection^e^	T, >9.3	11.67	(1.14–119.55)^f^	Case‐control study nested in a small cohort/case series. Peak TTV‐load in patients with infection were higher than in patients without.
Maggi, '18	99/235	CMV viremia	D0‐10 post Tx	C, 1	1.5	(1.0–2.3)^g^	Lower TTV‐load during 1‐year follow‐up in CMV DNA negative versus positive patients. Proposed threshold of 3.45 log to detect CMV reactivation.
Strassl, '18	31/72^h^	V/B/F	19–98 days pre infection	C, 1	1.23	(1.04–1.45)	Proposed threshold of 3.1*10^9 c/ml to predict infection. Higher TTV‐levels in samples taken before (severe/bacterial) infection.
Uhl, '20	40/45^i^	V/B/F	Time of infection	C, 1	1.016	(0.876–1.180)	The authors also report no association in analyses with the TTV‐load taken 1 month before infection (OR: 1.075, CI: 0.921–1.25) and infection causing fever (OR: 0.932, CI:0.724–1.199).
Fernández‐Ruiz, '19	128/221^j^	V/B/F/P	M1 post Tx	T, >3.15	2.88	(1.13–7.36)^k^	Proposed threshold of 3.15 and 4.16 log for infection and immunity related adverse events. Cases did not have higher TTV‐load at D0 and D7, and higher loads at M1, 3 and 6.
Fernández‐Ruiz, '20	54/205	BK viremia	M1 post Tx	T, >5.01	7.61	(2.09–27.70)^l^	This study contains an additional analysis on the cohort described above, for BKV viremia as outcome.
Gore, '20	40/666	V/B/F (death)	Hospital visit (1–20 yr post Tx)^m^	C, 1	1.26	(1.07–1.48)	Analysis stratified on time found an association >2 years after Tx. Time to outcome was similar in no, low, medium and high TTV‐load groups.
Jaksch, '18	28/143^n^	V/B/F	3mo intervals	C, Max	5.05	(2.94–8.67)	The authors report a higher cumulative frequency of infection in patients with a maximum TTV‐level >9.5 log in a 3 months window^o^.
Nordén, '17	nd/98^p^	V/B/F	Time‐varying (M3‐24)	C, 1	0.98	(0.87–1.11)	Analyses stratified on time (1–3, 3–6, 6–12 and 12–24 months post LuTx) showed no association between TTV‐load and any infection.
van Rijn, '21	105/389	BKV viremia	Time‐varying (M0‐12)	C, 1	1.03	(1.03–1.04)	The authors report similar outcomes in the analysis with CMV viremia as outcome (HR 1.01,CI: 1.01–1.01).
Frye, '19	19/34	V/B/F	D0, M2, 4, 6, 8, 10, 12	M		‐	No difference in TTV‐load at D0 and M2, and higher TTV‐load in patients with infection at the other timepoints.
Handala, '19	17/116	BKV viremia	D0, M1, 2, 3	M		‐	No difference in TTV‐load between patients with and without outcome at the timepoints. No correlation between TTV‐load and BKV DNA load at M3.
Herrmann, '18	23/115^q^	BKV viruria	Hospital visit, 0.6–34 yr post Tx	M		‐	In LiTx, they found a correlation between BKV DNA in urine and TTV DNA in serum, and higher TTV‐load in patients without BKV viruria than with.
Ruiz, '19	41/63	CMV viremia	During and pre infection	M		‐	Higher TTV‐load and cumulative TTV DNA during and before CMV infection and disease. No correlation between TTV‐load and CMV DNA during infection^r^.
Solis, '19	28/63^s^	BKV viremia	D0, M1, 3, 6, 12, 24	C, 0.2		^t^	No difference in TTV‐load at any timepoint between patients with and without BKV viremia during 2 year follow‐up, except at 6 months.

^a^ V: viral, B: bacterial, F: fungal, P: parasitic.

^b^ TTV exposure C: continuous, and step load increase in Log10. T: TTV load threshold, M: median or mean TTV load compared in patients with and without outcome.

^c^ OR: odds ratio, HR: hazard ratio.

^d^ Numbers from analysis with one event per patient.

^e^ Timing of TTV load sampling in control group is not reported.

^f^ OR not reported, approximated from reported test accuracy numbers. Large confidence interval due to very small groups.

^g^ It is unclear whether the reported OR is crude or adjusted for CMV prophylaxis, CMV negative serostatus and tacrolimus level at mo1.

^h^ Table 1 in the publication reports 22 with infection, the text reports 31 patients with infection and a total of 41 infections. In addition, the analysis allows for multiple outcomes per patient.

^i^ The analysis allowed for multiple infections per patient, 119 infectious episode occurred during the study period.

^j^ Sample size of the number of cases and total cohort. The sample size and number of cases at the month 1 timepoint is not reported.

^k^ Adjusted for delayed graft function and serum albumin at month 1.

^l^ Reported HR, adjusted for recipient age, hypertensive nephropathy and DCD donor.

^m^ Start of follow‐up time was set at hospital visit.

^n^ The authors allowed for multiple events per patients, events occurring within 1 mo were counted as 1 event.

^o^ Which 3 mo window is used to calculate the cumulative frequency is not reported.

^p^ The number of patients with outcome is reported only per periodic interval.

^q^ Only patients with detectable TTV loads were included (115/136).

^r^ The definition of cumulative DNA is not reported.

^s^ Cohort composed of 50 BKV viruric patients +16 non BKV‐viraemic patients, of which 63 had detectable TTV loads, and 28 were BKV viremic.

^t^ No OR reported or numbers from which the OR may be approximated.

As for TTV‐load, many studies found higher loads associated with a higher chance of infection. Multiple studies report significant ORs or HRs larger than 1 for various sample times, and corresponding to a continuous increase in TTV‐load,^21^, ^26^, ^28^, ^29^, ^30^ or a threshold.^14^, ^23^, ^24^ Other studies found a higher TTV‐load in patients with infection, compared to patients without infection.^16^, ^17^, ^32^ Four studies concluded no infection association was found in their population. Two of those, of which one measured the TTV‐load at the time of infection and the other at varying times after transplantation,^3^, ^13^ found no association with viral/bacterial/fungal infections. Another found a significant yet clinical irrelevant association with BKV and CMV infection,^4^ and the fourth found no difference in TTV‐load between patients with and without BKV infection on different timepoints after transplantation.^25^

### 3.5 TTV‐load as marker of rejection

Fifteen studies included rejection outcomes (Table 3).^3^, ^4^, ^5^, ^11^, ^12^, ^15^, ^17^, ^18^, ^19^, ^20^, ^23^, ^26^, ^28^, ^31^, ^32^ Most rejection outcomes were comparable across studies, as most were defined as biopsy‐proven rejection, based on the relevant international guidelines (Banff criteria for KTx,^33^ International Society for Heart and Lung Transplantation guidelines for LuTx,^34^ and the Rejection Activity Index score for LiTx^35^), or as rejection diagnosed based on clinical suspicion. Exact definitions differed, as some studies distinguished antibody mediated, acute or cellular rejection, and one LuTx study included chronic lung allograft dysfunction (CLAD) as outcome.^20^

**TABLE 3:** Summary of evidence – rejection outcomes

Study	Cases/total	Outcome^a^	TTV timepoint	Exposure^b^	OR/HR^c^		Other findings
Blatter, ‘18	17/48^d^	ACR	2wks post Tx	T, >median	0.16	(0.01–0.63)^e^	No association found at 6 weeks and 6 months post Tx.
Doberer, ‘19	11/37	BPR	2wks pre biopsy	C, 1	0.8	(0.64–1.00)^f^	Similar results in subanalyses without borderline lesions. Proposed threshold of 1.5*10^6 c/ml.
Doberer, ‘20	19/82	BPR	Day of biopsy	C, 1	0.91	(0.85–0.97)	Similar results for different subtypes of rejection.
Görzer, ‘17	20/47^g^	CLAD	Day of CLAD	T, >7	0.12	(0.03–0.50)^h^	Proposed threshold of 7.0 log. Similar findings with the TTV‐load taken 50–20 days before CLAD, and lower a TTV‐load found during and before CLAD.
Schiemann, ‘17	46/715	ABMR	Day of rejection^i^	C, 1	0.91	(0.87–0.96)^j^	Similar results were found in subanalyses with patients subject to protocol biopsy.
Strassl, ‘19	33/113	BPR	±1.5 mo pre biopsy^k^	C, 1	0.90	(0.84–0.97)	Similar results using only the earliest biopsy. Proposed threshold of 6 log to detect rejection.
Fernández‐Ruiz, ‘19	11/221^l^	AR	Before Tx	C, 1	0.69	(0.49–0.97)	Lower loads were found in patients with versus without outcome.
Jaksch, ‘18	11/143	ACR	3 month intervals	C, Min	0.48	(0.26–0.88)^m^	Similar results are shown for CLAD. A higher cumulative frequency of ACR is shown for TTV‐load <7.0 log in a 3 months window^n^.
Simonetta, ‘17	nd/29	BPAR	Before Tx	T, Pos	0.01	(0.001–0.092)^o^	Unadjusted Kaplan Meier curve also showed a lower cumulative incidence of rejection in TTV positive at D0 patients.
Solis, ‘19	28/63^p^	AMR	Before Tx	T, >3.4	0.14	(0.04–0.43)^q^	A similar result was found at M1, with a threshold of 4.2 log.
van Rijn, ‘21	88/389	AR	Time‐varying	C, 1	0.74	(0.71–0.76)	No other analyses are performed for rejection.
Blatter, ‘20	21/51	ACR	Before Tx, 1, 2, 3, and 5 wks (ratio)^r^	M		‐	No association between TTV‐load (ratios) and ACR or graft dysfunction.
Frye, ‘19	13/13^s^	BPR	D0‐M1	T, x1/10^t^		^u^	A sensitivity and specificity of 0.74 and 0.99 is shown for detecting BPR^s^. Findings were similar for different outcome definitions.
Nordén, ‘17	nd/98^v^	AR	Time‐varying (M3‐24)	C		^u^	Analyses stratified on time (1–3, 3–6, 6–12 and 12–24 months post Tx) showed no association between the TTV‐load and the number of acute rejections.
Ruiz, ‘19	19/63^v^	BPR	During rejection	M		‐	TTV‐load was similar patients with versus without BPR, and lower during clinical versus subclinical/no rejection, and moderate versus mild rejection.

Abbreviatopns: ACR, Acute cellular rejection; ABMR, antibody mediated rejection; AR, Acute rejection; BPAR, Biopsy proven acute rejectionc; BPR, biopsy proven rejection; CLAD, chronic allograft dysfunction; HR, hazard ratio; OR, odds ratio.

^a^ TTV exposure C: continuous, and step load increase in Log10. T: TTV load threshold, M: median or mean TTV load was compared in patients with and without outcome.

^b^ Number taken from figure.

^c^ Inverse of reported OR from supplement S2.

^d^ Adjusted for ATG as induction and previous SOT.

^e^ Total includes 27 controls.

^f^ Approximated from test accuracy numbers.

^g^ Median of 4.4 (ABMR+) and 6.6 (ABMR‐) yr after Tx.

^h^ Numbers from univariate analysis.

^i^ Median of 127 d (105–174) post‐Tx.

^j^ Number reported in supplement S6.

^k^ Numbers reported in text, however the numbers reported in the table differ slightly.

^l^ Which 3 month window is used to determine the TTV‐load is not reported.

^m^ Adjusted for age, gender, HBV, HCV, HIV serostatus, underlying disease, number of immunosuppressive drugs used, hepatic encephalopathy, and presence of DSA's.

^n^ A cohort composed of 50 BKV viruric patients +16 non BKV‐viremic patients, of which 63 had detectable TTV‐load, and 28 were BKV viremic.

^o^ Inverse of reported HR.

^p^ TTV‐load ratios were used as exposure.

^q^ The test accuracy is calculated on a biopsy proven rejection group.

^r^ Author definition of threshold: 10‐fold decrease of the individual TTV‐DNA levels within 1 month independently of the relative TTV‐DNA level.

^s^ No OR reported or numbers from which the OR can be approximated.

^t^ The number of patients with outcome is reported only per periodic interval.

^u^ ORs not reported.

^v^ 19 patients with 20 episodes of rejection.

As for TTV‐load, most studies reported an inverse association between the TTV‐load and rejection. However, one study found no association between TTV‐load and rejection, with TTV as time‐varying covariate, nor with stratifying on time.^3^ Another study was unable to replicate the results found in their previous analysis.^12^ The remaining 13 studies found lower odds or hazards for increasing TTV‐loads,^4^, ^5^, ^11^, ^15^, ^18^, ^19^, ^20^, ^23^, ^26^, ^28^, ^31^ or lower TTV‐loads in patients with rejection compared to without rejection.^17^, ^32^

### 3.6 Meta‐analysis

**FIGURE 4:**
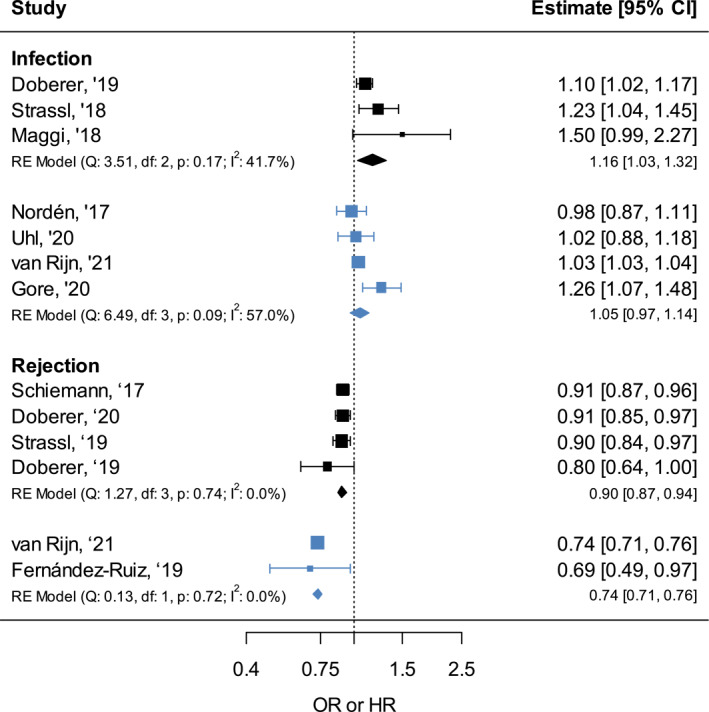
Meta‐analysis of the odds ratios (ORs) (black) and hazard ratios (HRs) (blue) for infection and rejection after solid organ transplant (SOT) associated with a TTV‐load increase on a continuous scale, per 1 log, for infection and rejection

For both effect measures, infection and rejection, a meta‐analysis was performed in those studies that used 1 log TTV‐load increase as definition of the exposure. For the calculations, a random‐effects model was used to accommodate the heterogeneity of the included studies. By pooling the results, an overall OR of 1.16 (95% CI: 1.03–1.32) and HR of 1.05 (95% CI: 0.97–1.14) was found for infection, and an overall OR of 0.90 (95% CI: 0.87–0.94) and HR of 0.74 (95% CI: 0.71–0.76) for rejection (Figure 4). Thus, meta‐analysis found a higher chance of infection and a lower chance of rejection with increasing TTV‐load. Meta‐analyses restricted to only adults, only KTx or only TTV PCR data obtained with in‐house primers showed similar results (Supplementary S3).

Residual heterogeneity was moderate for the overall HR for infection (I^2^: 57.0%, Cochran's Q *p*‐value: 0.09). Analysis restricted to only in‐house primers had an *I* ^2^ of 0.0% and Cochran's Q *p*‐value of 0.67, which indicates that the heterogeneity may be due to pooling of the in‐house and R‐GENE primers. The other overall analyses did not show significant heterogeneity. Assessment of publication bias through a funnel plot was not possible due to the small number of studies (<10) included per analysis.

## 4 DISCUSSION

Torque teno virus is proposed as an attractive and useful marker to predict complications of over‐ and under‐immunosuppression, infection and rejection respectively, and guide immunosuppressive dosing in SOT care. By systematically searching and reviewing the current literature, we identified multiple studies exploring the association between TTV‐load and post‐SOT infection and rejection. Despite various concerns on study quality and design, most studies report positive associations between TTV‐load and infection, and negative associations between TTV‐load and rejection. Some studies did not find significant associations, but studies that reported associations in opposite, unexpected directions were not found. Meta‐analysis of a subset of the studies confirmed the presence of these associations.

The association with TTV‐load is more apparent for the rejection outcomes. Meta‐analysis found a pooled OR of 0.90 (95% CI: 0.87–0.94) and HR of 0.74 (95% CI: 0.71–0.76) per 1 log TTV‐load increase. This result indicates that a patient experiencing a 5 log TTV‐load increase, which is not at all uncommon after SOT, has an OR of 0.59 or HR of 0.22 for developing rejection, compared to no TTV‐load increase. For the infection outcomes, the TTV‐load association was less apparent, with the meta‐analysis finding a pooled OR of 1.16 (95% CI: 1.03–1.32) and HR of 1.05 (95% CI: 0.97–1.14). Not all individual studies were able to find a significant association between high TTV‐load and infection, for outcomes varying from a combination of viral, bacterial and fungal infections^3^, ^13^ and for BKV infection.^25^

A stronger association with rejection might be expected taken into consideration the pathogenic mechanisms underlying rejection and infection. Torque teno virus is thought to be a marker of ‘immune function’, and rejection a direct consequence of under‐immunosuppression. In contrast, complications of over‐immunosuppression are more often multi‐factorial, and, in case of infection, include (partially) immune‐independent factors like reactivation of latent pathogens in the recipient, and exposition to new pathogens from the environment or from the donor organ.^36^, ^37^ Therefore, TTV may well turn out to be a better predictor of rejection than of infection.

While the TTV‐load level is hypothesised to mirror ‘immune function’, it is not known what part of the immune system it mirrors precisely. Generally speaking, cellular immunity likely controls the number of virus producing cells, and humoral immunity likely neutralises circulating virus, but this has not been established yet. Furthermore, the mechanism of TTV‐replication is not understood, although studies suggest that replication occurs in activated peripheral blood mononuclear cells (PBMC).^38^, ^39^, ^40^ Therefore, the effect of immunosuppressive drugs on TTV‐load as well as the relationship between TTV‐load and different immunomonitoring markers require further study. Outside immunology, also the relevance of detecting particular (combinations of) TTV‐genotypes after transplantation needs to be addressed in this context.

The discovery and identification of the predictive effect of TTV‐load is still a new research field—all but one studies included in this systematic review were published from 2017 onwards. Many angles remain to be further explored. We recommend future research to focus on the clinical usage and application of TTV‐load measurement, for instance to identify ideal timepoints of sample collection, estimate the added clinical value over already known predictors of infection and rejection, and to publish full prediction models enabling external evaluation of the performance of these models. In addition, it will be important to optimise and compare ways of expressing TTV‐load, for example, as absolute or logarithmic values, or as the load increase/decrease calculated over a fixed period of time after transplantation.

Several limitations could have influenced the results and conclusions of the systematic review. For a start, the designs were quite diverse across studies. Study populations often differed across SOT type, and included adult as well as pediatric transplantations. Furthermore, TTV loads were measured using different PCRs, at different timepoints and with variable follow‐up. Moreover, different outcome definitions were used. However, by performing meta‐analyses restricted to these specific study populations and TTV detection methods, we learned that the statistical heterogeneity caused by these factors is likely small, because the additional analyses showed similar results. Second, the observed risks of bias observed for a considerable number of studies could have created deflated, inflated, or even spurious results. A similar thing can be said about the small and exploratory nature of most studies, analysing many timepoints and trying different angles in the statistical analyses, which could have caused inflation of the reported effect sizes through selective reporting, which was observed in the risk of bias assessment.^41^ The extent of the publication bias could not be determined statistically due to the small number of studies. Larger studies with pre‐specified analyses are definitively needed to confirm the results. All together, these limitations could influence the reported effect size both positively and negatively, and therefore the pooled results from the meta‐analyses and conclusions should be interpreted with caution.

In conclusion, in recent literature TTV has been presented as a compelling candidate to anticipate infection and rejection after SOT. This systematic review shows that the current literature does not offer definitive confirmation of its predictive ability due to quality concerns and lack of validation, nor prediction for individual patients that may be readily used by treating physicians. However, the association between TTV‐load and complications is apparent in the reviewed studies and provides a good starting point for studies on the basic principles of TTV‐replication and immunity, as well as for larger studies into TTV as marker of infection and rejection after SOT.

## AUTHOR CONTRIBUTIONS

AL van Rijn, JI Rotmans and MCW Feltkamp designed the study. AL van Rijn and R Roos performed the screening, data extraction and bias evaluation, and MCW Feltkamp resolved differences in agreement. AL van Rijn prepared the manuscript, which was checked and shaped by R Roos, FW Dekker, JI Rotmans and MCW Feltkamp by providing critical feedback.

## CONFLICT OF INTEREST

AL van Rijn, MCW Feltkamp and JI Rotmans are consortium partners of the EU‐funded TTV Guide Tx trial (https://cordis.europa.eu/project/id/896932) and will receive financial support as participating center, to carry out the trial. No funding was received for the preparation of this manuscript.

## Supporting information

Supplementary Material S1, S2, and S3

## Data Availability

The data that support the findings of this study are available from the corresponding author, AvR, upon reasonable request.
