# Supplementary material for: Torque teno virus load as marker of rejection and infection in solid organ transplantation – A systematic review and meta‐analysis
Source: Rev Med Virol. 2022 Sep 3;33(1):e2393. doi: 10.1002/rmv.2393 (PMC10078304; doi:10.1002/rmv.2393)
Supplement: Supplementary file 1 — Supplementary Material S1, S2, and S3 [file RMV-33-0-s001.docx]

# Supplementary material

## 1. Search strategy

**Databases**

- PubMed
- Embase
- Web of Science
- COCHRANE Library
- Emcare
- Academic Search Premier

**Search term**

(("Torque teno virus"[Mesh] OR "Torque Teno Virus"[tw] OR "Torque Teno Vir*"[tw] OR "Transfusion Transmitted Vir*"[tw] OR "TTV"[tw] OR "TT V"[tw] OR "TT Vir*"[tw] OR "SEN virus"[tw] OR "SEN vir*"[tw] OR "SENV"[tw] OR "SEN V"[tw] OR (("alpha*"[tw] OR "teno vir*"[tw]) AND "torque"[tw]) OR "Anelloviridae"[Mesh] OR "Anello*"[tw]) AND ("SOTx"[tw] OR "solid organ*"[tw] OR "Organ Transplantation"[Mesh] OR "Organ Transplant*"[tw] OR "Organ Graft*"[tw] OR "Organ Allotransplant*"[tw] OR "Organ Allograft*"[tw] OR "Heart Lung Transplant*"[tw] OR "Heart Transplant*"[tw] OR "Hepatic Transplant*"[tw] OR "Kidney Transplant*"[tw] OR "Renal Transplant*"[tw] OR "Liver Transplant*"[tw] OR "Lung Transplant*"[tw] OR "Pancreas Transplant*"[tw] OR "Pancreatic Transplant*"[tw] OR "Heart Lung Graft*"[tw] OR "Heart Graft*"[tw] OR "Hepatic Graft*"[tw] OR "Kidney Graft*"[tw] OR "Renal Graft*"[tw] OR "Liver Graft*"[tw] OR "Lung Graft*"[tw] OR "Pancreas Graft*"[tw] OR "Pancreatic Graft*"[tw] OR "Heart Lung Allotransplant*"[tw] OR "Heart Allotransplant*"[tw] OR "Hepatic Allotransplant*"[tw] OR "Kidney Allotransplant*"[tw] OR "Renal Allotransplant*"[tw] OR "Liver Allotransplant*"[tw] OR "Lung Allotransplant*"[tw] OR "Pancreas Allotransplant*"[tw] OR "Pancreatic Allotransplant*"[tw] OR "Heart Autotransplant*"[tw] OR "Hepatic Autotransplant*"[tw] OR "Kidney Autotransplant*"[tw] OR "Renal Autotransplant*"[tw] OR "Liver Autotransplant*"[tw] OR "Lung Autotransplant*"[tw] OR "Pancreas Autotransplant*"[tw] OR "Pancreatic Autotransplant*"[tw] OR "Heart Lung Allograft*"[tw] OR "Heart Allograft*"[tw] OR "Hepatic Allograft*"[tw] OR "Kidney Allograft*"[tw] OR "Renal Allograft*"[tw] OR "Liver Allograft*"[tw] OR "Lung Allograft*"[tw] OR "Pancreas Allograft*"[tw] OR "Pancreatic Allograft*"[tw] OR "Hepatic Autograft*"[tw] OR "Kidney Autograft*"[tw] OR "Renal Autograft*"[tw] OR "Liver Autograft*"[tw] OR "Lung Autograft*"[tw] OR "Pancreas Autograft*"[tw] OR "Pancreatic Autograft*"[tw] OR (("Transplants"[mesh] OR "Transplantation"[mesh] OR "Transplantation Immunology"[Mesh] OR "Transplant*"[tw] OR "Graft*"[tw] OR "Allotransplant*"[tw] OR "Allograft*"[tw] OR "Autotransplant*"[tw] OR "Autograft*"[tw]) AND ("Organ"[tw] OR "Organs"[tw] OR "Heart Lung"[tw] OR "Heart"[tw] OR "Hepatic"[tw] OR "Kidney"[tw] OR "Liver"[tw] OR "Lung"[tw] OR "Pancreas"[tw] OR "Pancreatic"[tw] OR "Hands"[tw] OR "Hearts"[tw] OR "Kidneys"[tw] OR "renal"[tw] OR "Livers"[tw] OR "Lungs"[tw])) OR "Transplant Recipients"[Mesh] OR "Recipient*"[tw] OR "Tissue Donors"[Mesh] OR "Tissue Donor*"[tw] OR "Graft Rejection"[Mesh] OR "Graft Reject*"[tw] OR "reject*"[tw] OR "transplant*"[tw] OR "graft*"[tw] OR "allotransplant*"[tw] OR "allograft*"[tw] OR "autotransplant*"[tw] OR "autograft*"[tw] OR "xenotransplant*"[tw] OR "xenograft*"[tw]) AND english[la] NOT (("Case Reports"[ptyp] OR "case report"[ti]) NOT ("Review"[ptyp] OR "review"[ti] OR "Clinical Study"[ptyp] OR "trial"[ti] OR "RCT"[ti])))

## 2. Medication per study

| **Study** | **Population** | **Induction immune suppresion** | **Maintenance immune supression** | **Antibacterial prophylaxis** | **Antiviral prophylaxis** |
| --- | --- | --- | --- | --- | --- |
| Blatter et al. 2018 | Children | Site-specific | CNI (Tac), Antimetabolites (MMF) and Steroids | n.m. | Standard-of-care antiviral prophylaxis |
| Blatter et al. 2020 | Adults | Anti-IL2 (Basiliximab) + MPS or ATG + MPS | CNI (Tac), Antimetabolites (MMF) and Steroids | n.m. | Antiviral prophylaxis |
| Doberer et al. 2019 | Adults | Anti-IL2 (Basiliximab) or ATG or Anti-IL2 + anti-CD20 (Rituximab) | CNI (Tac), Antimetabolites (MMF) and Steroids | Trimethoprim/sulfamethoxazole | Valganciclovir on indication |
| Doberer et al. 2020 | Adults | Anti-IL2 (Basiliximab) or ATG or Anti-IL2 + anti-CD20 (Rituximab) | CNI (Tac), Antimetabolites (MMF) and Steroids | Trimethoprim/sulfamethoxazole | Valganciclovir on indication |
| Fernández-Ruiz et al. 2019 | Adults | Anti-IL2 (Basiliximab), ATG or no induction | CNI (Tac) with/without mTOR-inhibitor (Sirolimus), Antimetabolites (MMF or AZA) and Steroids | Cefazolin/ciprofloxacin + Trimethoprim/sulfamethoxazole or pentamidine | Valganciclovir or ganciclovir on indication |
| Fernández-Ruiz et al. 2020 | Adults | Anti-IL2 (Basiliximab), ATG or no induction | CNI (Tac) with/without mTOR-inhibitor (Sirolimus), Antimetabolites (MMF or AZA) and Steroids | Cefazolin/ciprofloxacin + Trimethoprim/sulfamethoxazole or pentamidine | Valganciclovir or ganciclovir on indication |
| Frye et al. 2019 | n.m. | Anti-IL2 (Basiliximab) | CNI (Tac) with/without Everolimus, Antimetabolites (MMF) and Steroids | antibiotic prophylaxis | n.m. |
| Gore et al. 2020 | Adults | n.m. | CNI (Tac or CsA), mTOR-inhibitors, Antimetabolites (MMF or AZA) and/or Steroids | n.m. | n.m. |
| Görzer et al. 2014 | Adults + Children | Induction, no induction or not available | CNI (Tac or CsA), Antimetabolites (MMF) and Steroids | n.m. | n.m. |
| Görzer et al. 2017 | Adults | Alemtuzumab, ATG or no induction | CNI (Tac or CsA), Antimetabolites (MMF) and Steroids | n.m. | n.m. |
| Handala et al. 2019 | Adults | Anti-IL2 (Basiliximab) or ATG | CNI (Tac or CsA), Antimetabolites (MMF) and Steroids | n.m. | n.m. |
| Herrmann et al. 2018 | Adults | n.m. | CNI (Tac or CsA), Antimetabolites (MMF) and/or Steroids (in some cases Everolimus or Sirolimus) | n.m. | n.m. |
| Jaksch et al. 2018 | Adults | Alemtuzumab, ATG or no induction | CNI (Tac), Antimetabolites (MMF) and Steroids | n.m. | n.m. |
| Maggi et al. 2018 | Adults | Anti-IL2 (Basiliximab), ATG or no induction | CNI (Tac or CsA), Antimetabolites (MMF) and Steroids | n.m. | Anti-CMV prophylaxis |
| Nordén et al. 2017 | Adults | ATG with MPS | CNI (Tac or CsA), Antimetabolites (MMF) and Steroids | Trimethoprim/sulfamethoxazole | Valganciclovir on indication |
| Ruiz et al. 2019 | Adults | Anti-IL2 (Basiliximab) or no induction | CNI (Tac or CsA), Antimetabolites (MMF) and/or Steroids | Trimethoprim/sulfamethoxazole | Valganciclovir on indication |
| Schiemann et al. 2017 | Adults | Induction or no induction | CNI (Tac or CsA) or mTOR-inhibitor, Antimetabolites (MMF), Steroids and/or Belatacept | n.m. | n.m. |
| Simonetta et al. 2017 | Adults + Children | n.m. | n.m. | n.m. | n.m. |
| Solis et al. 2019 | Adults | Anti-IL2 (Basiliximab) or ATG | CNI (Tac or CsA), Antimetabolites (MMF) and Steroids | n.m. | n.m. |
| Strassl et al. 2018 | Adults | Anti-IL2 or ATG | CNI (Tac), Antimetabolites (MMF) and Steroids (some switched to CsA, Everolimus or Belatacept) | Trimethoprim/sulfamethoxazole | Valganciclovir on indication |
| Strassl et al. 2019 | Adults | Anti-IL2 or ATG | CNI (Tac), Antimetabolites (MMF), Steroids and/or Belatacept | Trimethoprim/sulfamethoxazole | Valganciclovir on indication |
| Uhl et al. 2020 | Children | n.m. | CNI (Tac or CsA) or mTOR-inhibitor (Sirolimus), Antimetabolites (MMF or AZA) and/or Steroids | n.m. | n.m. |
| van Rijn et al. 2021 | Adults | Anti-IL2 (Basiliximab) or alemtuzumab | CNI (Tac or CsA), Antimetabolites (MMF) and Steroids | n.m | n.m. |

*n.m: not mentioned*

## 3. Stratified meta-analyses

**Only adult populations:**

**Only pediatric populations:** Pooling not possible, only one study (Uhl et al, 2020)

**Only using in-house primers:**

**Only using the R-GENE primers:** Pooling not possible, only two studies (Fernandez-Ruiz et al 2019, and Gore et al 2020)

**Only kidney transplantation populations:**

**Only lung/liver transplantation:** Pooling not possible, only two studies (Maggi et al 2018, and Norden et al 2017)
